# Correction of hallux abducto valgus by scarf osteotomy. A ten-year retrospective multicentre review of patient reported outcomes shows high satisfaction rates with podiatric surgery

**DOI:** 10.1186/s13047-022-00546-3

**Published:** 2022-06-02

**Authors:** Sharon Clee, George Flanagan, Julian Pavier, Ian Reilly

**Affiliations:** 1 Department of Podiatric Surgery, Northamptonshire Healthcare NHS Foundation Trust (NHFT), Northampton, UK; 2 Department of Podiatric Surgery Nottinghamshire Healthcare NHS Foundation Trust, Nottingham, UK; 3 Private Practice, The Park Hospital, Nottingham; and Circle Nottingham, Nottingham, UK; 4 Private Practice, Three Shires Hospital Northampton, Northamptonshire, UK; 5 Private Practice, Ramsay Woodlands Hospital, Kettering, Northamptonshire UK

**Keywords:** Hallux abducto valgus, Hallux valgus, Bunion, Bunion/surgery, Hallux valgus/surgery

## Abstract

**Background:**

Corrective surgery for hallux abducto valgus is one of the most performed elective procedures in foot and ankle practice. Numerous methods of surgical correction have been reported within the literature, with varying clinical and patient reported outcomes. This study reviews the patient experience and outcomes in five podiatric surgery centres using the scarf diaphyseal osteotomy.

**Method:**

Patient reported outcome measures (PROMs) were captured using the Patient Satisfaction Questionnaire 10 (PSQ-10), part of the PASCOM-10 podiatric surgery audit tool. PROMs were collated across five hospital sites over a 10-year period.

**Results:**

Of 1351 patients reported during the period, 1189 had complete retrospective data. The most common patient aim of surgery was ‘no/less pain’ reported in 70% of patients. 96.8% of patients reported their original foot complaint as ‘better’ or ‘much better’ after surgery. 92.8% of patients reported their expectations had been met with 96.6% reporting they would have surgery again under the same conditions. 98.5% of patients noted that the risks, complications, and expectations had been discussed prior to surgery. The most common complication was metatarsal fracture (4.6%).

**Conclusion:**

The scarf osteotomy (with or without an Akin phalangeal osteotomy) consistently showed high patient satisfaction with low complication rates using PSQ-10 and this information can be used as part of the pre-operative consenting process. Patient expectations for surgery were often achieved, which may be attributed to the pre-operative work up of the patient. Further investigation into this correlation is suggested.

**Level of clinical evidence:**

IV (retrospective review).

**Supplementary Information:**

The online version contains supplementary material available at 10.1186/s13047-022-00546-3.

## Introduction

Hallux abducto valgus (HAV), the bunion deformity, is one of the most common presentations in foot and ankle practice [1, 2]. It is a complicated multiplanar pathology and is characterised by lateral deviation and valgus rotation of the hallux and medial deviation of the first metatarsal [1, 3, 4]. It has been reported that approximately 23% of adults aged between 18 and 65 years have the deformity, which increases to 35.7% of the population above 65 years of age [3]. It has a higher female predilection and can often result in a decreased quality of life [5]. HAV is often intractable despite conservative care, ultimately requiring a surgical correction for many patients after a failure of conservative care [1, 2]. Over a hundred different surgical solutions have been proposed throughout the literature with varying outcomes [6, 7]. Schrier et al. [8] suggested that up to a third of patients operated on for HAV may be dissatisfied with the outcome of their surgery (though the paper they attribute this value to in fact demonstrated good patient outcomes). Patient reported outcomes are now crucial in capturing and evaluating treatment effectiveness.

### Bunion surgery using the scarf (+/− Akin) osteotomy

Although no single surgical procedure has shown superiority, the scarf osteotomy is a popular choice in HAV correction due to its versatility in treating mild, moderate, and even severe HAV deformity [9–13]. The ‘scarf’ term is derived from a carpentry method where two pieces of wood are joined together with the long ends overlapping. This creates stability via a construct which can resist tension and compression forces [14]. The scarf osteotomy was traditionally be performed via translation (see Figs. 1 and 2) or rotation of the osteotomy fragment, the latter technique utilised for deformities with higher intermetatarsal angle (IMA) [15]. Lopez et al [7] describe the translation and rotation procedure or ‘trotation’ scarf osteotomy (see Figs. 3 and 4). The scarf osteotomy is often undertaken alongside an Akin osteotomy (a phalangeal, closing adductory wedge osteotomy) to augment the hallux abductus component of the HAV deformity [16].

**Fig. 1:**
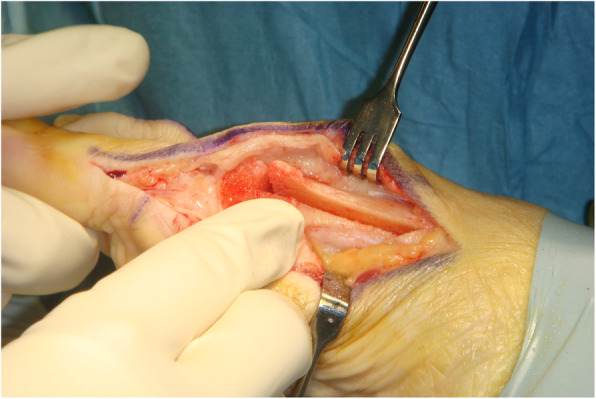
Translation scarf – direct lateral translation of the capital fragment

**Fig. 2:**
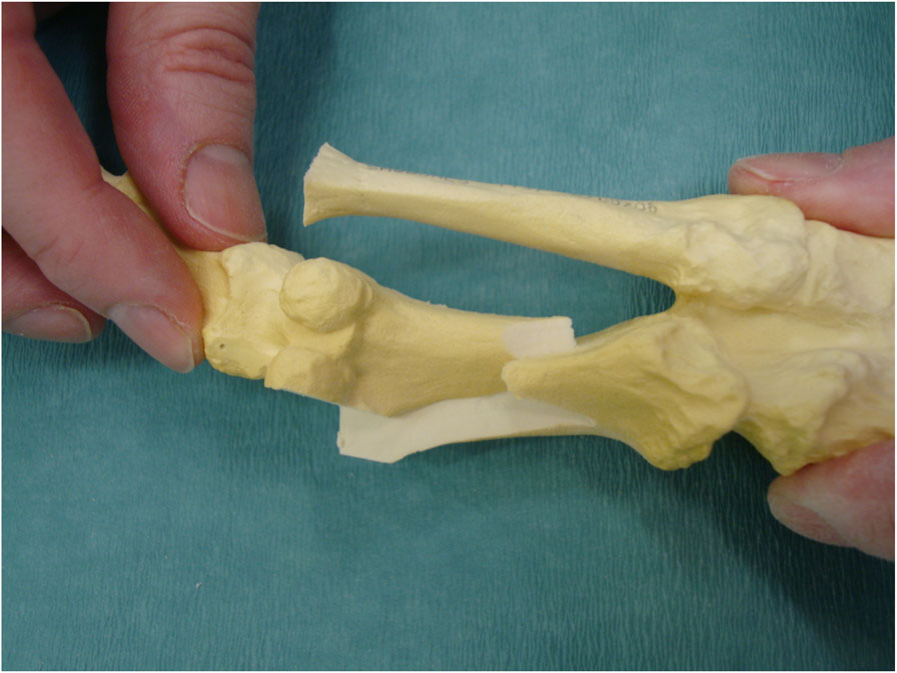
Translation scarf (saw bone)

**Fig. 3:**
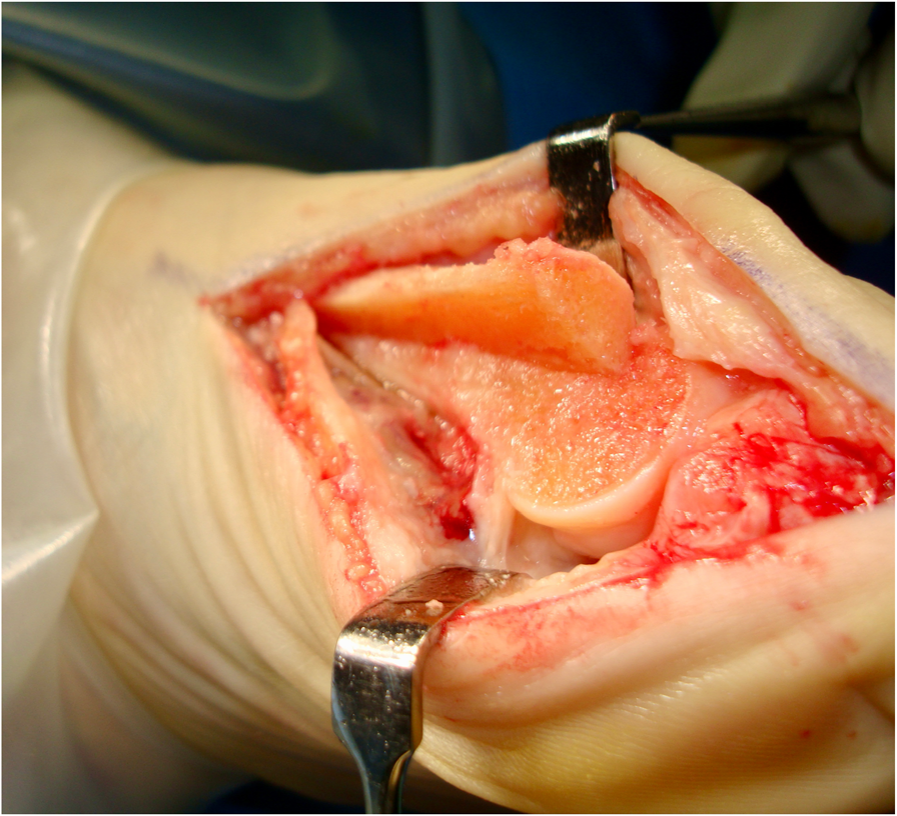
Trotations scarf – translation and lateral rotation of the capital fragment

Fixation of the scarf osteotomy may vary from surgeon to surgeon but most commonly, two points of internal fixation are used. In more recent years, cannulated compression screws have been used where AO cortical screws were previously the norm. Some surgeons choose to use a Kirschner (K) wire and a screw as their chosen two points of fixation, e.g., Lopez et al [7]. The Akin osteotomy may be fixed with either a single threaded K wire, a staple, or screw, as the intact lateral hinge is utilised as the second point of fixation.

### PASCOM-10 and the PSQ-10

Foot health outcome measurement tools can be used to improve service delivery by collating and evaluating parameters such as pain, foot function, footwear, and mobility [17]. The Podiatric Audit of Surgery and Clinical Outcome Measurement system (PASCOM) was developed by the College of Podiatry in 1997 with the updated PASCOM-10 introduced to the podiatric surgery profession in the UK in 2010 [18]. It provides a structured framework in which to collate and compare data relating to the characteristics, outcome, and patient experiences of foot surgery [19]. It is a web-based database of podiatric procedures and outcomes which allows for retrospective reviews [20].

PASCOM-10 has three domains with the first encompassing the surgical treatment, the second relating to post-operative sequelae with the final section housing the patient satisfaction questionnaire (PSQ-10, 18]. The PSQ-10 questionnaire has ten questions (see Additional file 1) with the first asking the patient an open-ended question about their expectations of their surgery. The following nine answers are scored with a maximum of 100 points. Higher scores are an indication of high levels of satisfaction, whilst a minimum score of zero indicates the opposite [17, 19]. The PSQ-10 has not undergone formal validation, although it has reliably demonstrated that satisfaction do no change over time [17, 19].

**Fig. 2:**
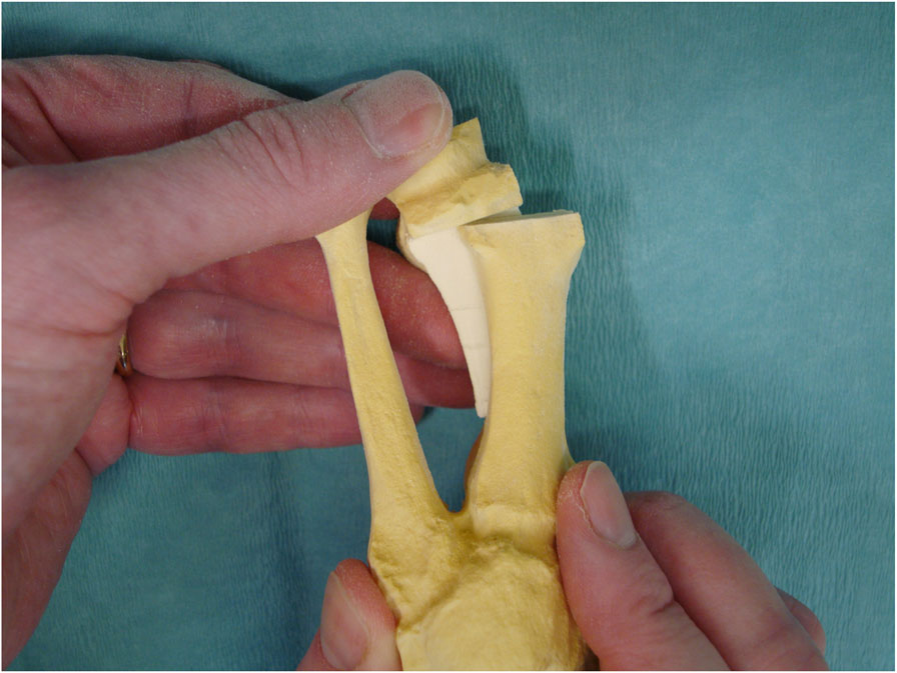
Trotation scarf (saw bone)

Digital PASCOM-10 files are created when a patient is listed for surgical intervention and data is collated on the surgical information, complications, satisfaction, and patient reported outcome measure (PROM) scores. Activity is entered by clinical or administrative staff on the day of surgery and then at a 6 month post-surgical review [21], at which point the PSQ-10 questionnaire is completed together with the recording of any post-operative sequelae. If a patient fails to attend the 6-month review, there is an option for the questionnaire to be emailed to the patient, but often these episodes are lost to follow up.

This aim of this paper was to review the subjective evaluation of the patients’ outcome and recorded clinical sequelae across five podiatric surgery centres over a 10 year period. Incidental but valuable data relating to pre-operative surgeon/patient conversation was also captured.

## Methods

### PASCOM data

A retrospective review of patient outcomes for all patients who underwent bunion surgery utilising a scarf osteotomy was performed using surgical audit data generated by and extracted from the PASCOM-10 database. The database has a built-in reporting system which can identify the appropriate patients relevant to a specific line of enquiry. The raw data collected from PASCOM-10 reported for any scarf procedure (or variant) performed either on its own or in combination with an Akin osteotomy, uni- and bilaterally. Patients who had adjunctive procedures at the same time, e.g., concurrent hammer toe surgery, were excluded in case this skewed the data (n = 1192). Additional analysis was performed using Microsoft Excel spreadsheet (Microsoft® Excel® for Microsoft 365 MSO (16.0.14326.20706) 64-bit). Reports were assembled from dates between 01/01/2010 (the inception of the current, on-line iteration of PASCOM-10) and 31/12/2019. Data from 2020 was not included as there had been a significant reduction in services due to COVID-19.

Five centres were included in the study which consisted of both NHS and private practices. No limitation was placed on the operating surgeon for the search. The NHS services are podiatric surgery training centres, and the procedures were performed by podiatric consultants, podiatric registrars, or surgical trainees under direct consultant supervision. Post-operatively, patients were reviewed in clinic for an initial dressing at two weeks with a further dressing review a week or fortnight later. Patients were then advised regarding rehabilitation exercises and return to activities before being reviewed six months following the original surgery.

In Northampton, consent for the recoding and use of PASCOM-10 data is done capturing the following at a pre-surgery health questionnaire, completed by the patient:

#### Section 12: surgery audit – helping us improve our clinical outcomes

To the patient: I understand that the clinician wishes to maintain my data on PASCOM-10, a web-based surgery audit system (with high level password protection). This will be used for anonymised data regarding my treatment outcomes and PASCOM information is held within my patient records. The purpose for using this database is to monitor the results and benefits of my treatment. I understand that my data will be anonymised so that no personal information can be retrieved. I can view my electronic PASCOM-10 record if I wish. When my data is used in reports my name will not appear and I cannot be identified. I also consent to be contacted by the podiatric surgery department to take part in any future research or evaluation. If I decline this does not affect my normal rights to have treatment, nor will I be treated differently in any way. I give my permission for my information to be used in this way:

…………………………………… Signature of patient/date

## Results

A total of 1603 scarf osteotomies (all variants) were performed using 4557 pieces of orthopaedic hardware to fixate, of which of which 3491 (76.6%) were a standard AO screw or equivalent. There was a total of 1351 patients in the cohort with 1227 females (90.83%, average age 59.13, standard deviation [SD] 13.51) and 124 males (9.17%, average age 59.43 SD 15.37) who had undergone a scarf HAV correction with or without an Akin osteotomy. The greatest percentage of females who underwent surgery were in the 60–79 age group accounting for 47.38% of the total cohort. Males in the same age range accounted for 5.10% of the population. The average outcome data score was 89.68 out of 100 (SD 9.64). Regrettably, not all episodes were fully captured on the PASCOM-10 database: 1189 (88%) patients completed the PSQ-10 questionnaire but 162 (12%) were lost to follow up (Table 1).

**Table 1:** Age demographics for patients

Age	Male	Male %	Female	Female %
0–19	1	0.07	0	0.00
20–39	15	1.11	131	9.70
40–59	34	2.51	411	30.42
60–79	69	5.10	640	47.38
80+	5	0.37	45	3.33
Total	**124**	**9.17**	**1227**	**90.83**

Of those patients who completed PSQ-10, 1177 (98.9%) patients identified their expectations for the surgery in Q1 of the questionnaire: ‘briefly state what you expected to gain from treatment’. The responses were collated into Microsoft Excel (Microsoft 365) which could then be arranged into four common themes. Some patients mentioned more than one expectation but a reduction in pain (70%) was the biggest goal for patients, followed by a ‘better looking’ or ‘straighter foot’ (25.5%) and better footwear fit/choices (25%). Only 12% of the patients who answered were looking for a return to sports or the ability to function better on their foot following their bunion correction. This may be partly due to the age demographics with over 74% of patients aged over 55 who are perhaps less likely to partake in active sports. A summary of the PSQ-10 Q1 themes can be found in Table 2.

**Table 2:** Responses from Q1 from PSQ-10 questionnaire – What do you expect to gain from treatment?

	No/less pain	Better looking/ straighter foot	Easier footwear	Walking better / return to sports
**Total responses**	819	303	295	143
**%**	70%	25.7%	25%	12%

A summary of answers (as per PASCOM, to one decimal place, which gives rounding errors in questions 6 and 9) to other questions, condensed for the brevity of this article, is listed in Table 3.

**Table 3:** A summary of the PSQ-10 questionnaire answers

Question No.	Answer	No/%
2. Were the risks and complications explained to you before you had your operation?	Yes	1171 (98.5%)
	No/not sure	12 (1%)
	Not stated	6 (0.5%)
3. Did you know what to do if you had a problem after your operation?	Yes	1164 (97.9%)
	No/not sure	14 (1.2%)
	Not stated	11 (0.9%)
4. Did you have a problem after surgery?	No	913 (76.8%)
	Yes - minor	225 (18.9%)
	Yes - major	44 (3.7%)
	Not stated	7 (0.6%)
5. After your operation, how effective was your pain control	Pain control did not work	69 (5.8%)
	Some discomfort	670 (56.3%)
	Minimal or no pain	435 (36.6%)
	Not stated	15 (1.3%)
6. When could you get back into your shoes?	2 weeks	209 (17.6%)
	4 weeks	354 (29.8%)
	6 weeks	294 (24.7%)
	8 weeks	234 (19.7%)
	12 weeks	46 (3.9%)
	By 6 months	32 (2.7%)
	6+ months	13 (1.1%)
	Not stated	7 (0.6%)
7. Do you still have discomfort from your original foot condition?	No	507 (42.6%)
	Occasional twinge	540 (45.4%)
	Standing long time	91 (7.7%)
	When standing	17 (1.4%)
	Yes, even at rest	26 (2.2%)
	Not stated	8 (0.7%)
8. How would you describe your original foot condition since treatment?	Much better	950 (79.9%)
	Better	201 (16.9%)
	The same	21 (1.8%)
	A little worse	7 (0.6%)
	Deteriorated	4 (0.3%)
	Not stated	6 (0.5%)
9. Would you be prepared to have surgery performed under the same conditions again?	Yes	1148 (96.6%)
	No	32 (2.7%)
	Not stated	9 (0.8%)
10. Were the original expectations that you stated at the beginning met?	Yes	1103 (92.8%)
	In part	71 (6.0%)
	No	9 (0.8%
	Not stated	6 (0.5%)

Post-operative complication data was gathered from the in-built reporting tool. In 75.1% (964 of the cohort), no sequalae were observed. A detailed breakdown of post-surgical sequalae can be observed in Table 4, as per PASCOM, to one decimal place, which gives rounding errors.

**Table 4:** Most frequently occurring post-operative sequalae

Sequalae (out of 1351 patients)	Number	%
No observed sequalae	964	75.1
Metatarsal fracture	62	4.6
Joint pain & stiffness > 3 months	53	4.1
Scar hypertrophy / keloid	26	2.0
Swelling beyond 4 months	25	1.9
Pain at surgical site > 6 weeks	23	1.8
Transfer metatarsalgia	21	1.6
Fixation problems requiring removal	20	1.5
Infection suspected / not proven	18	1.4
Stitch abscess / suture reaction	12	0.9
Wound dehiscence	10	0.8
Surgery failed e.g., recurrence, hallux varus)	9	0.7
Infection proven	7	0.5
Deep vein thrombosis	7	0.5
Excessive post-operative pain (first 72 hours)	5	0.4
Patient non-compliance	5	0.4
Motor power weakness	3	0.2
Bone union delay	2	0.2
Medication side effect	1	0.1
Callus or IPK formation	1	0.1
Haematoma	1	0.1

## Discussion

PROMs are becoming an integral component of health care practice, and which should encompass items that are relevant to the patient [22]. Baumhauer et al [23] showed that outcome factors regarded by patients as important for foot/ankle complaints differ from factors judged by physicians. It was the intention at the conceptual stage of the PSQ-10 questionnaire to capture and explore the patient’s perception of their treatment rather than assessing the outcome from a clinician’s point of view [19]. Maher and Tollafield [18] proposed that a PSQ-10 threshold score of 75 or above can be for isolated HAV correction. In their paper, they presented a mean PSQ-10 score of 86.5 for patients who underwent a scarf osteotomy. The timing of obtaining a PROM is dependent on many variables [5]. Rudge & Tollafield [19] asked centres to send questionnaires to patients 6 months after surgery and thus the consensus within UK podiatric surgical units is that PROMs have continued to be gathered at this point following treatment. Further work is currently being undertaken by the Northampton team to identify if PASCOM-10 outcome scores change over time.

From this study it was found that a total of 1151 patients (96.8% of the cohort) felt that their foot condition was either ‘better’ (16.9%) or ‘much better’ (79.9%) following surgery. This is consistent with the findings of Crevoisier et al. [24], and Spruce et al [25], who each identified that 94% of their cohort felt they had improved following a scarf osteotomy. 86% of Crevoisier’s cohort said they would undergo the same procedure again whilst Kerr et al. [26] reported that only 74.3% of their patients who underwent a scarf osteotomy would. These findings are lower than the results of this study which showed 96.6% would proceed with the same surgery in the future. Importantly, over 92% of our patients felt that their expectations had been fully met following the scarf osteotomy with 6% of those answering feeling that they had been partly met. The reasons for this warrant further study. Less than 1% felt that their expectations for surgery had not been met following the surgical procedure. Managing expectations to surgery is crucial in a consent to surgery, and value may be found in analysing the outliers.

Metatarsal troughing, delayed union, rotational malunion, recurrence of deformity and infection were all identified as post-operative complications following a scarf osteotomy by Coetzee [27]. Metatarsal fracture was the most common post-operative complication affecting 4.6% of our cohort but may reflect differences in the recording of sequela by the five units. In discussion between authors, the Nottingham units included any minor osteotomy movement as a fracture. Fracture was not shown to be a factor in the study performed by Lipscombe et al. [28] who only had two complications: a traumatic neuroma and avascular necrosis requiring an arthrodesis of the first metatarsophalangeal joint. A midterm experience of scarf osteotomy by Samaras et al [11] highlighted 15 out of 70 ft with complications which included seven recurrences, one deep vein thrombosis (DVT), four cases of complex regional pain syndrome, three intra-operative fractures and three cases of failure of osteosynthesis. Smith and colleagues [10] reported metatarsal fractures: these were intraoperative in two cases whilst two stress fracture were seen at three and 7 months post operatively. They didn’t however identify in which metatarsal the stress fractures occurred. Barouk [29] observed 3% of his study to be at risk of metatarsal fracture.

Joint pain and stiffness were recorded in 53 (4.1%) of patients in this study which is much less than that found by Crevoisier et al [24] who found that first metatarsophalangeal joint motion worsened in 7% of their patients who underwent a scarf osteotomy in isolation, and in 6.7% of patients who underwent an additional Akin osteotomy [22]. Our analysis identified 26 patients (2%) who acknowledged a problem with their scar at their six-month review which differs from Ieong et al. [30] who found that 31% of their patients had experienced symptoms at the scar site when they were reviewed at a minimum of 1 year post operatively.

There were no reported incidences of complex regional pain syndrome (CRPS) in our study. Conversely, Samaras et al [11] and Deenik et al [31] noted rates of 5 and 10.6% respectively. Seven cases of DVT were highlighted in our study equating to 0.5% of the cohort. As noted above, Samaras et al. [11] documented only one case of DVT in their 70 patients undergoing a scarf osteotomy. Their patients were noted to have been prescribed low molecular weight heparin (LMWH) for 3 weeks despite being allowed to immediately weight bear in a heel wedge shoe. This seems to contradict the thoughts of Jameson et al. [32] who suggested that patients in the U.K. undergoing bunion surgery had no clear risk factors for venous thromboembolism (VTE) and VTE prophylaxis had no benefit in ambulatory cases. Harrison and Walker [33] also proposed that patients who are ambulatory after day case surgery were not considered to be at high risk of VTE and forefoot surgery should be considered low risk.

As with any audit and data collection, the results are only as good as the information inputted. Consequently, the post-operative sequalae shown in this study may be underestimated. Some post-operative dressings were performed by specialist nurses or health care assistants in the private sector who may not have had access to the PASCOM-10 database, and this may therefore represent an under reporting of incidences, such as stitch abscesses. Many of the common complications are seen early in the post-operative phase whilst the main post-operative concerns are inputted into the PASCOM-10 software at 6 months following surgery when the PSQ-10 questionnaire is completed. In the future it would be prudent to record all sequalae as and when they occur, rather than waiting until the final 6 month review. The link between sequela and final outcome is not well delineated using PASCOM-10 and it is unclear from the data set if those patients that experienced a complication of surgery (e.g., hallux varus) were or were not satisfied with their overall result. Future studies would need to build in systems to cross reference these factors.

Both NHS units are training centres for podiatric surgery trainees and registrars. It was not possible from the collated data to unpick whether the outcome varied between surgeon grades (and therefore by extrapolation, experience). This would be a useful area to unpick in future studies but would require separate data to be prospectively gathered using PASCOM-10.

## Conclusion

Although the figures presented are mid-term results, the authors believe that the data gives a good indication of the outcomes and post-operative sequelae that may affect patients undergoing a scarf (with or without an Akin) osteotomy for HAV correction as performed by podiatric surgeons. This should enable a more informed consenting process with our patients in the future. The data suggests good outcomes for the procedure in a wide cohort of patients, across five podiatric centres, where much of the surgery is performed by non-consultant grades. This study has also highlighted the importance of inputting post-operative sequalae as they occur, rather than waiting until the final review, to capture a more accurate and comprehensive overview of any concerns raised by both patient and clinician.

Patient expectations of surgery were often achieved in the centres studies which may be attributed to the pre-operative work up of the patient. Further investigation into this correlation is suggested.

## Supplementary Information

**Additional file 1.** PSQ-10 Questionnaire.

## Data Availability

Data and material is anonymised data on PASCOM-10 but raw data is available from the authors on resonable request.
