# Supplementary material for: Correction of hallux abducto valgus by scarf osteotomy. A ten-year retrospective multicentre review of patient reported outcomes shows high satisfaction rates with podiatric surgery
Source: J Foot Ankle Res. 2022 Jun 2;15:44. doi: 10.1186/s13047-022-00546-3 (PMC9159929; doi:10.1186/s13047-022-00546-3)
Supplement: Supplementary file 1 [file 13047_2022_546_MOESM1_ESM.docx]

PASCOM Patient Satisfaction Questionnaire (P.S.Q 10)

**Name :**	**Operation date :**

1. Briefly state what you expected to gain from treatment, in the space provided below.

2. Were the risks and possible complications of surgery explained to … you, before you had your operation?

- ☐ Yes
- ☐ Not sure
- ☐ No

3. Did you know what to do if you had a problem after your operation?

- ☐ Yes
- ☐ Not sure
- ☐ No

4. Did you have a problem after your operation?
(if you answered 'No', then go straight to question 5 overleaf, otherwise answer questions 4a – 4d below)

- ☐ No
- ☐ Yes, a minor problem
- ☐ Yes, a major problem

4a. When you had your problem, how did you seek help?

- ☐ I waited until my next appointment and raised it then
- ☐ I telephoned for help and was given an earlier appointment
- ☐ I called the Podiatrist out
- ☐ I contacted the Podiatrist who gave me advice over the telephone
- ☐ I attended my local hospital casualty department
- ☐ Other (state)

4b. If you rang the Podiatry Department, when did you call?
(Ignore this question if you did not telephone for help)

- ☐ 9 am - midday
- ☐ Midday to 5 pm
- ☐ 5 pm to 9 am

4c. If you rang the Podiatry Department, how did you find the speed of response? (Ignore this question if you did not telephone for help)

- ☐ Slow to respond
- ☐ Satisfactory
- ☐ Fast to respond

4d. Overall how would you say your problem was dealt with?

- ☐ Poorly
- ☐ Cannot say as problem is still being managed
- ☐ Satisfactorily
- ☐ Excellently

Please turn over and complete the questions on the other side

5. After your operation, how effective was your pain control?	☐	Pain control did not work
	☐	Some pain but I coped
	☐	Minimal or no pain
6. When could you get back into your shoes?	☐	By two weeks
	☐	By four weeks
	☐	By six weeks
	☐	By eight weeks
	☐	By six months
	☐	Six months and over
7. Do you still have discomfort from your original foot condition?	☐	Yes, even whilst at rest
	☐	Yes, when standing
	☐	Yes, when standing for long periods
	☐	Just occasional twinges
	☐	No discomfort
8. How would you describe your original foot condition since treatment?	☐	Deteriorated
	☐	A little worse
	☐	The same
	☐	Better
	☐	Much better
9. Would you be prepared to have surgery performed under the same conditions again?	☐	Yes
	☐	No
10. Were the original expectations that you stated at the beginning of this questionnaire met?	☐	Yes
	☐	In part
	☐	No

Thank you for your co-operation in filling in this questionnaire.

The results of our patient surveys are used to improve the quality of service that we provide to our patients and will remain anonymous.

Return your questionnaire to this address:
